# Qiliqiangxin attenuates hypoxia‐induced injury in primary rat cardiac microvascular endothelial cells via promoting HIF‐1α‐dependent glycolysis

**DOI:** 10.1111/jcmm.13572

**Published:** 2018-03-04

**Authors:** Yanyan Wang, Xueting Han, Mingqiang Fu, Jingfeng Wang, Yu Song, Yuan Liu, Jingjing Zhang, Jingmin Zhou, Junbo Ge

**Affiliations:** ^1^ Department of Cardiology Shanghai Institute of Cardiovascular Diseases Zhongshan Hospital Fudan University Shanghai China; ^2^ Department of Cardiology Zoucheng Hospital Affiliated Hospital of Jining medical university Jinan Shandong China

**Keywords:** cardiac microvascular endothelial cells, glucose metabolism, HIF‐1α, hypoxia injury, qiliqiangxin

## Abstract

Protection of cardiac microvascular endothelial cells (CMECs) against hypoxia injury is an important therapeutic strategy for treating ischaemic cardiovascular disease. In this study, we investigated the effects of qiliqiangxin (QL) on primary rat CMECs exposed to hypoxia and the underlying mechanisms. Rat CMECs were successfully isolated and passaged to the second generation. CMECs that were pre‐treated with QL (0.5 mg/mL) and/or HIF‐1α siRNA were cultured in a three‐gas hypoxic incubator chamber (5% CO_2_, 1% O_2_, 94% N_2_) for 12 hours. Firstly, we demonstrated that compared with hypoxia group, QL effectively promoted the proliferation while attenuated the apoptosis, improved mitochondrial function and reduced ROS generation in hypoxic CMECs in a HIF‐1α‐dependent manner. Meanwhile, QL also promoted angiogenesis of CMECs via HIF‐1α/VEGF signalling pathway. Moreover, QL improved glucose utilization and metabolism and increased ATP production by up‐regulating HIF‐1α and a series of glycolysis‐relevant enzymes, including glucose transport 1 (GLUT1), hexokinase 2 (HK2), 6‐phosphofructokinase 1 (PFK1), pyruvate kinase M2 (PKM2) and lactate dehydrogenase A (LDHA). Our findings indicate that QL can protect CMECs against hypoxia injury via promoting glycolysis in a HIF‐1α‐dependent manner. Lastly, the results suggested that QL‐dependent enhancement of HIF‐1α protein expression in hypoxic CMECs was associated with the regulation of AMPK/mTOR/HIF‐1α pathway, and we speculated that QL also improved HIF‐1α stabilization through down‐regulating prolyl hydroxylases 3 (PHD3) expression.

## 1 INTRODUCTION

Coronary artery disease (CAD) is the most common cardiovascular disease, characterized by insufficient oxygen supply.^1^ It is known that cardiac microvascular endothelial cells (CMECs) directly mediate the exchange of substance and energy between microcirculation and myocardial tissue and thus serve as the essential elements responsible for maintaining the normal myocardial tissue metabolism.^2^ Hypoxia‐induced CMECs injury is considered as an initiating process and pathological basis of various cardiovascular diseases,^3^ and protecting CMECs from hypoxia insult might thus be an important therapeutic strategy for treating various cardiovascular diseases.^4^ Hypoxia can insult various endothelial cell biological activities such as cell growth, survival, migration and energy metabolism, which may lead to angiogenesis impairment, redundant reactive oxygen species (ROS) generation, mitochondrial dysfunction and energy metabolism disturbance to some extent.

Hypoxia‐inducible factor‐1α (HIF‐1α) is an important oxygen balance regulator which plays critical roles in the transportation and utilization of oxygen.^5^ HIF‐1α mediates hypoxia‐induced adaptive changes in various cell types by activating various downstream target genes responsible for oxygen delivery, glucose metabolism,^6^ angiogenesis,^7^ cell proliferation and survival. Under hypoxic conditions, the uptake of glucose and glycolytic flux is increased mostly due to the HIF‐1α‐dependent up‐regulation of genes encoding glucose transporters GLUT1/4,^8^ glycolytic enzymes (eg HK2, PDK1 and LDHA) and mitochondrial proteins (eg BNIP3 and COX4I2).^9^ Besides delivering nutrients and oxygen to organs and tissues, endothelium also plays a critical role in regulating tissue glucose metabolism via HIF‐1α transcriptional regulation of GLUT1 expression.^10^, ^11^ Previous studies showed that up‐regulated HIF‐1α activity was linked with reduced the apoptosis of various cell types including cardiomyocytes and human umbilical vein endothelial cells (HUVECs).^12^, ^13^ On the contrary, suppressed HIF‐1α expression was related to reduction in angiogenesis and glycolysis, increase in ROS production and induction of apoptosis or death of endothelial cells, cancer cells and other cell types.^14^, ^15^, ^16^, ^17^, ^18^ Taken together, HIF‐1α is a key regulator of endothelium survival under hypoxic condition, and up‐regulation of HIF‐1α might be a key method of protecting endothelial cells from hypoxic injury.

Qiliqiangxin (QL), a traditional Chinese medicine compound preparation, is composed of 11 Chinese herbal medicines (such as astragalus, ginseng and aconite). Protective effects of QL were demonstrated by a multicenter randomized double‐blind clinical study including 512 heart failure patients.^19^ It was also reported that QL could improve cardiomyocyte metabolism and inhibit cardiomyocyte apoptosis.^20^, ^21^, ^22^, ^23^ Our previous work found that QL could promote cardiac angiogenesis and up‐regulate HIF‐1α expression in failing heart and hypoxic CMECs via NRG‐1/ErbB‐PI3K/Akt/mTOR pathway, thus promoting cardiac angiogenesis.^24^, ^25^ However, it remains elusive whether QL could improve glucose metabolism in CMECs under hypoxic insult and inhibit CMECs apoptosis via modulating HIF‐1α activity and related target genes. This study addressed this issue and investigated whether QL could affect glucose metabolism and cell apoptosis in hypoxic CMECs via HIF‐1α‐dependent mechanisms. Furthermore, we investigated whether QL up‐regulates HIF‐1α protein expression by regulating post‐translationally hydroxylated of HIF‐1α or via the AMPK/mTOR/HIF‐1α pathway.

## 2 MATERIALS AND METHODS

### 2.1 Primary rat CMECs isolation and culture

Two‐week‐old male SD rats (30 ± 5 g) were purchased from the Shanghai Jie Esprit Experimental Animal Co. Ltd. (Shanghai, China). Isolation and culture of primary CMECs were performed with explant method, and the procedures were as follows: the rats were killed by cervical dislocation and the heart was quickly excised and rinsed with 4°C pre‐colding PBS. After cutting off the upper part of the great vessels and atrial tissues, the epicardium and endocardium were gently torn. The remaining ventricular tissues were cut into 1 mm^3^ small pieces and plated onto a 10‐cm culture dish pre‐coated with in 1 mL of foetal bovine serum (FBS). Tissues were then cultured in 37°C, 5% CO2 incubator for 4 hours and for another 48 hours in high‐glucose Dulbecco's modified Eagle's medium (high‐glucose DMEM) (Gibco, #1791920) supplemented with 10% FBS. After 72 hours, when the endothelial cells reached a confluency of 80%, the tissue pieces were removed and the cells were detached using 0.25% trypsin and passaged. The second generation of cells were used for experiments.

### 2.2 Small interfering RNA (siRNA) transfection

The second generation of endothelial cells were plated in 6‐well plates and cultured overnight. Then, the cells were transfected with HIF‐1α siRNA (100 nmol/L) or a negative control (NC) siRNA (100 nmol/L) using riboFECT™ CP Reagent and Buffer, according to the manufacturer's protocol. The sequence of the HIF‐1α siRNA was TCGACAAGCTTAAGAAAGA. Both the siRNA and transfection reagent were purchased from RiboBio Co., Ltd. (Guangzhou, China). Briefly, 10 μL siRNA/NC‐RNA were diluted in 120 μL 1 × riboFECT™ CP Buffer and 12 μL riboFECT™ CP Reagent, mixed fully and incubated for 20 minutes at room temperature. Then, this riboFECT™ CP mixture, together with 1858 μL DMEM, was added to each well and incubated at 37°C for 36 hours. Thereafter, cells were processed according to experimental protocols.

### 2.3 Hypoxia treatment and experimental group

In our previous study, we found that HIF‐1α expression peaked at 12 hours of hypoxia, so we performed our experiments at 12 hours of hypoxia in this study. Hypoxia was induced using a three‐gas hypoxia incubator chamber (5% CO_2_, 1% O_2_ and 94% N_2_). For drug intervention, cultured CMECs were pre‐treated with 0.5 mg/mL QL for 1 hour before hypoxia, which was prepared as in our previous study.^25^ Cells were randomly divided into following groups: NC group; Hypoxia group; Hypoxia + HIF‐1α siRNA group; Hypoxia + QL group; Hypoxia + QL + HIF‐1α siRNA group. Another group of CMECs was incubated with or without AICAR (1 mmol/L, AMPK activator) in the absence or presence of QL.

### 2.4 CCK‐8 cell proliferation assay

Cell proliferation level was examined using Cell Counting Kit‐8 (CCK‐8) according to the manufacturer's instructions (DOJINDO LABORATORIES, Shanghai, China). Briefly, after being transfected with HIF‐1α siRNA for 48 hours, CMECs were resuspended with 0.25% trypsin and reseeded in 96‐well plate (6 × 10^3^ cells), followed by hypoxia exposure for 12 hours. Then, the cells were incubated with 10 μL CCK‐8 staining solution for 2 hours at 37°C under normoxic conditions, and the absorbance (ODs) value at 450 nm was detected by microplate reader (Synergy™ H4; BioTek Instruments, Inc. USA) to calculate cell proliferation levels.

### 2.5 Caspase‐3 activity assay

Caspase‐3 is a key enzyme mediating apoptosis, the activity of which can reflect the apoptosis of endothelial cells. Caspase‐3 can catalyse substrate DEVD‐AFC (AFC: 7‐amino‐4‐trifluoromethyl coumarin) which emits blue light (λmax = 400 nm); upon cleavage of the substrate by CPP32 or related caspases, free AFC emits a yellow‐green fluorescence (λmax = 505 nm), which can be quantified using a fluorescence microtitre plate reader. By comparing the fluorescence strength of AFC using Caspase‐3/CPP32 Fluorometric Assay Kit, the activity of caspase‐3 was determined (BioVision, Inc., San Francisco, USA). After 12 hours of hypoxia, 80 μg cell lysates were dissolved in 50 μL chilled cell lysis buffer and 50 μL reaction buffer (2×, containing 10 mmol/L DTT). Then, 5 μL 1 mmol/L DEVD‐AFC substrate was added (final concentration of 50 μmol/L), and samples were incubated at 37°C for 1 hour. Finally, samples were read with a fluorometer equipped with a 400‐nm excitation filter and 505‐nm emission filter (Synergy™ H4; BioTek Instruments, Inc. USA).

### 2.6 Tube formation assay

Chilled liquid Matrigel (#354234; Corning Incorporated, New York, USA) was dispensed onto 24‐well plates (200 μL per well) and solidified in incubator at 37°C for 1 hour. Then, 500 μL CMECs (5.0 × 10^4^ cells per well), which have been transfected with HIF‐1α siRNA or control siRNA for 48 hours, were seeded onto the gel and cultured with DMEM containing QL (0.5 mg/mL) at 37°C in hypoxia incubator chamber for 12 hours. The enclosed networks of complete tubes from 3 randomly chosen fields were photographed under a microscope (Olympus, Tokyo, Japan). Finally, the tube formation images were analysed by the software WimTube Image Analysis (Ibidi GmbH, Germany).

### 2.7 Determination of the levels of VEGF secretion

After the cell culture, supernatants were collected by centrifugation at 300 g for 10 minutes and the levels of VEGF secretion were detected using a commercial rat VEGF ELISA kit (Multi Sciences, Hangzhou, China) according to the manufacturer's instructions and calculated as pg/mL protein.

### 2.8 Lactate dehydrogenase (LDHA) activity and glucose level

In order to estimate the level of glycolysis, cell culture supernatants were collected at the end of the experiment, and LDHA activity (Nanjing Jiancheng Bioengineering Institute, Nanjing, China) and glucose level (BioSino Bio‐Technology & Science Inc., Beijing, China) were measured according to the manufacturer instructions, respectively.

### 2.9 Measurement of intracellular ATP levels

After hypoxia and QL treatment, the intracellular ATP levels were measured using an ATPlite assay kit (Beyotime Biotechnology, Shanghai, China) according to the manufacturer's instruction. Luminescence was detected using a fluorescent plate reader (Synergy™ H4; BioTek Instruments, Inc. USA).

### 2.10 Detection of intracellular reactive oxygen species (ROS) levels

ROS generated in CMECs were assessed by dihydroethidium (DHE) staining which can freely go through the cell membrane into the cell and be dehydrogenated by superoxide anion to produce ethidium. Ethidium can be combined with RNA or DNA to produce red fluorescence (EX/EM = 300 nm/610 nm). After hypoxia for 12 hours, cells were washed by PBS 3 times and incubated with 10 μmol/L at 37°C for 30 minutes. Images were taken at a fixed exposure time and background intensity under an Olympus fluorescent microscope. For each group, 16 random high‐resolution fields (repeat 4 trials, 4 random fields for each trial) were randomly chosen and counted in a blinded manner. The relative DHE fluorescence intensity was detected and expressed as fold changes normalized to control group (set as 1) determined using ImageJ software, the similar method to calculate the fluorescence was used in previous studies.^26^, ^27^

### 2.11 Measurements of mitochondrial transmembrane potential (ΔΨm)

Changes in ΔΨm were detected using a dual‐emission potential‐sensitive probe, 5, 5′, 6, 6′‐tetra‐chloro‐1,1′,3,3′‐tetraethyl‐imidacarbocyanine iodide (JC‐1) staining kit (Beyotime Biotechnology, Shanghai, China). CMECs were seeded in 6‐well plate and transfected with HIF‐1α siRNA for 48 hours. After being treated with QL and subjected to hypoxia for 12 hours, cells were incubated with dyeing working fluid for 20 minutes at 37°C. For images of JC‐1 monomers, the wavelengths were green (Ex = 514 nm, Em = 529 nm) and for J‐aggregates were red (Ex = 585 nm, Em = 590 nm), and images from 5 randomly chosen fields were photographed under a microscope (Olympus). Then, the fluorescence intensity of aggregates and monomers was analysed using ImageJ software using the same method as DHE staining, and the ratio of aggregates/monomers fluorescence intensity was calculated as an indicator of mitochondrial transmembrane potential.

### 2.12 Total RNA extraction and quantitative RT‐PCR

Total RNAs were extracted using the TRIzol reagent (Invitrogen, Carlsbad, CA, USA). One microgram of total RNA was reverse‐transcribed to cDNA using a RT reagent kit (TaKaRa, Tokyo, Japan), which was then amplified with SYBR green dye on a CFX Connect ™ Real‐Time System (Bio‐Rad Laboratories, Inc., California, USA). The primer sequences are listed in Table 1. The relative quantification of mRNA levels was calculated with the software of the PCR system by standard 2^**−▵▵Ct**^ relative quantification method.

**Table 1:** Primer sequences for HIF‐1α and glucose metabolism‐related genes

Genes	Forward primer	Reverse primer
GLUT1	5′‐CTGGCTGCTGGATAGAATGAG‐3′	5′‐TGTTGGGAGTCAATGGTGTC‐3′
HIF‐1a	5′‐CTCCCTTTTTCAAGCAGCAG‐3′	5′‐CAGGTGTTTCTTGGGTAGGC‐3′
VEGF	5′‐TCACCAAAGCCAGCACATAG‐3′	5′‐TTTCTCCGCTCTGAACAAGG‐3′
PKM2	5′‐TCCCATTCTCTACCGACCTG‐3′	5′‐TTCAGTGTGGCTCCCTTCTT‐3′
LDHA	5′‐GTCAGCAAGAGGGAGAGAGC‐3′	5′‐CACTGGGTTTGAGACGATGA‐3′
β‐actin	5′‐GAAGTGTGACGTTGACATCCG‐3′	5′‐TGCTGATCCACATCTGCTGGA‐3′

### 2.13 Western blot analysis

The cells were washed with pre‐colding PBS, lysed in lysis buffer on ice and then centrifuged at 14 000 *g* for 20 minutes at 4°C. A total of 10‐24 μg of cell total protein were separated using 10% or 12% SDS‐polyacrylamide gel electrophoresis and then transferred to PVDF membranes (Millipore, Billerica, MA, USA). After being blocked with 5% bovine serum albumin (BSA) for 1 hour at room temperature, the membranes were incubated with specific primary antibodies overnight at 4°C. On the second day, membranes were then washed with TBST and further incubated with horseradish peroxidase (HRP)‐labelled goat anti‐rabbit IgG at room temperature for 2 hours. Finally, signal density of the immunoblots was performed with the SuperSignal West Pico Chemiluminescent Substrate (Thermo Fisher Scientific Inc., USA) and Gel Doc™ XR+ System (Bio‐Rad Laboratories, Inc.) and analysed by Image Lab software.

### 2.14 Statistical analysis

The data were analysed using the SPSS software package. For the comparison between 2 groups, the differences in the mean values were evaluated by Student's *t* test and the Mann‐Whitney U test. For the comparison among 3 or more groups, differences were determined by one‐way ANOVA with Bonferroni post hoc test. A value of *P* < .05 was considered to be statistically significant. All data were expressed as the means ± SD.

## 3 RESULTS

### 3.1 QL promoted CMECs proliferation and inhibited apoptosis under hypoxic condition via up‐regulation of HIF‐1α

**Figure 1:**
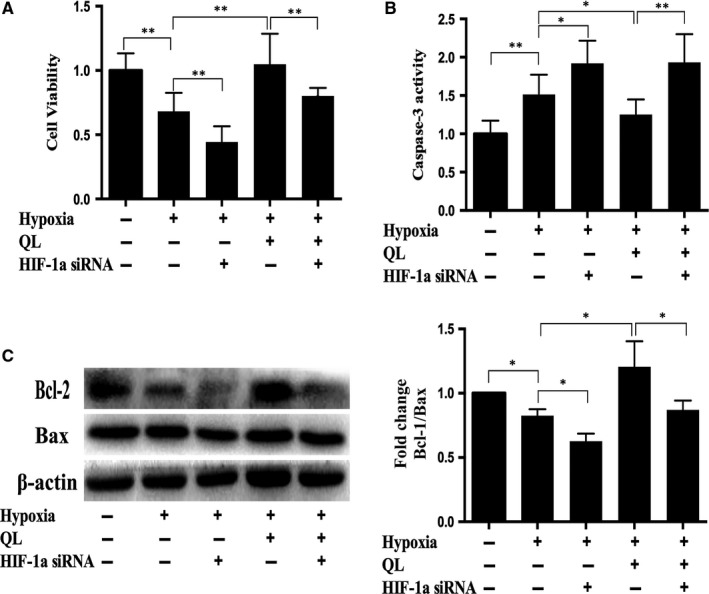
Effects of qiliqiangxin (QL) on cardiac microvascular endothelial cells (CMECs) proliferation and apoptosis. (A) QL promoted proliferation of hypoxic CMECs. Primary rat CMECs were exposed to hypoxia and/or QL for 12 h, and cell proliferation was detected using CCK‐8 assay (n = 4/group). (B) and (C) after hypoxia for 12 h, cell apoptosis was measured by caspase‐3 activity and Bcl‐2/Bax protein ratio (n = 4/group). Data were expressed as means ± SD. *P < .05; **P < .01

In order to examine the effects of QL on CMECs exposed to hypoxic injury and the role of HIF‐1α, the proliferation and apoptosis of hypoxic CMECs were analysed upon transfection of either HIF‐1a siRNA or control siRNA. CCK‐8 assay results demonstrated reduced cell proliferation post‐hypoxia, which could be reversed by QL treatment, but this beneficial effect of QL was significantly attenuated by HIF‐1α siRNA (Figure 1A). Meanwhile, CMECs apoptosis was evaluated by caspase‐3 activity and the Bcl‐2/Bax ratio. Caspase‐3 activity increased and the Bcl‐2/Bax ratio decreased under hypoxia, while QL significantly reduced hypoxia‐induced cell apoptosis, and this protective effect was partly abolished by cotreatment with HIF‐1α siRNA (Figure 1B,C). These data indicated that QL could promote CMECs proliferation and inhibit apoptosis under hypoxic condition, and these effects were at least partly due to HIF‐1α up‐regulation.

### 3.2 QL alleviated mitochondrial dysfunction and reduced ROS production under hypoxic condition in a HIF‐1α‐dependent manner

**Figure 2:**
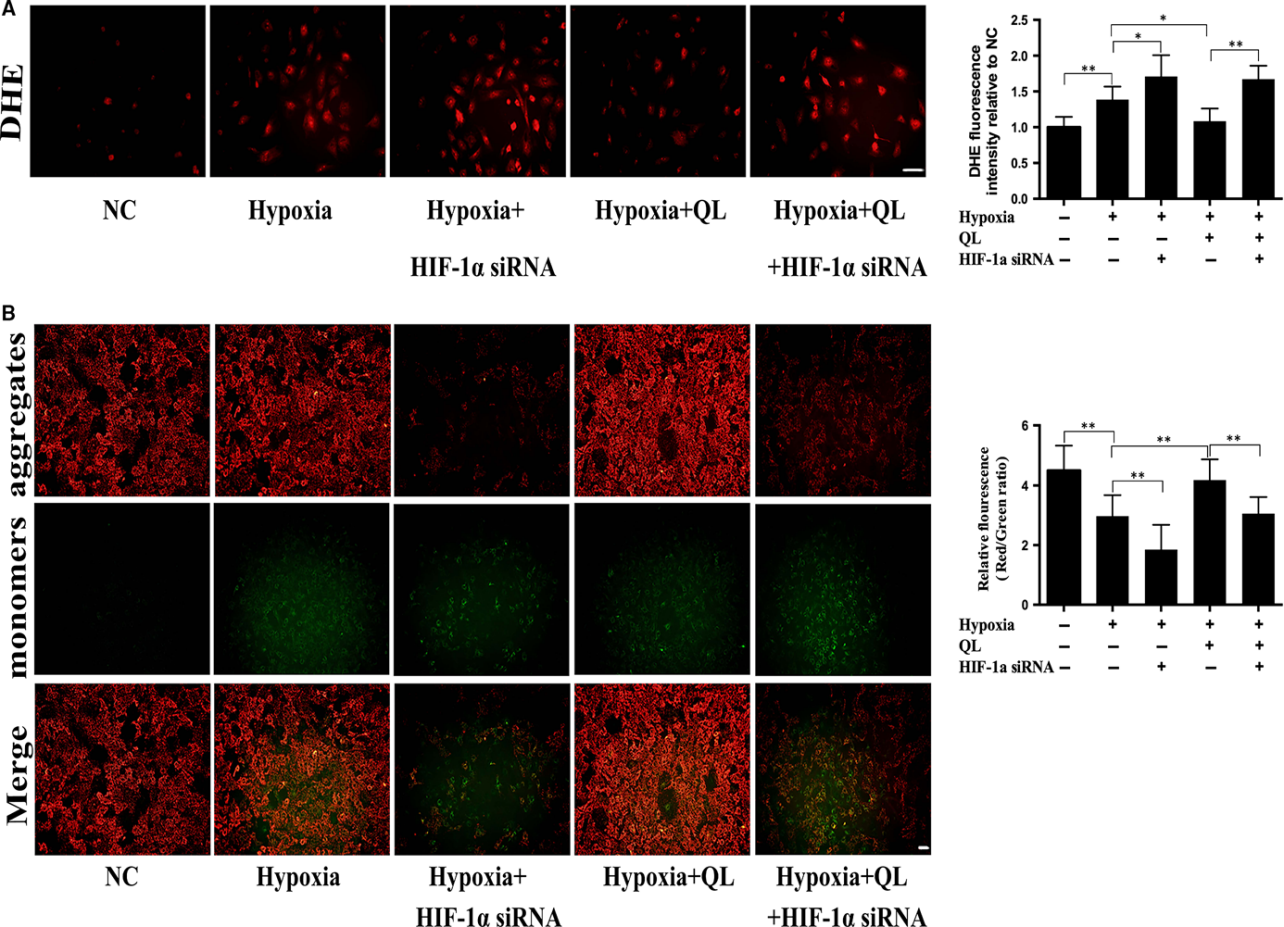
Effects of qiliqiangxin (QL) on ROS production and mitochondria membrane potential. (A) QL reduced ROS generation in cardiac microvascular endothelial cells (CMECs) exposed to hypoxia for 12 h. Dihydroethidium (DHE) staining was used to detect ROS level in CMECs (20×, Scale bar 50 μm) (n = 4/group). (B) QL attenuated mitochondria dysfunction in CMECs exposed to hypoxia for 12 h. Mitochondrial membrane potential was detected by JC‐1 staining (10×, Scale bar 100 μm) (n = 4/group). Data were expressed as means ± SD. *P < .05; **P < .01

As hypoxia induces mitochondria dysfunction, we used JC‐1 staining to measure mitochondrial transmembrane potential of CMECs. The results showed that the decreased ratio of aggregates/monomers in hypoxia group could be reversed by QL treatment, and this beneficial effect was partly abolished by HIF‐1α siRNA (Figure 2B). We also measured the intracellular ROS generation using the dihydroethidium probe, as hypoxia‐induced mitochondria dysfunction leads to ROS production. After hypoxia exposure for 12 hours, the intracellular ROS were increased in CMECs. Pre‐treatment with QL could decrease ROS production under hypoxic condition, while this beneficial effect was suppressed by HIF‐1α siRNA (Figure 2A). Taken together, these results suggested that QL could attenuate hypoxia‐induced mitochondrial dysfunction and decrease ROS production in hypoxic CMECs in a HIF‐1α‐dependent manner.

### 3.3 QL promoted CMECs angiogenesis under hypoxic condition via HIF‐1α/VEGF pathway

**Figure 3:**
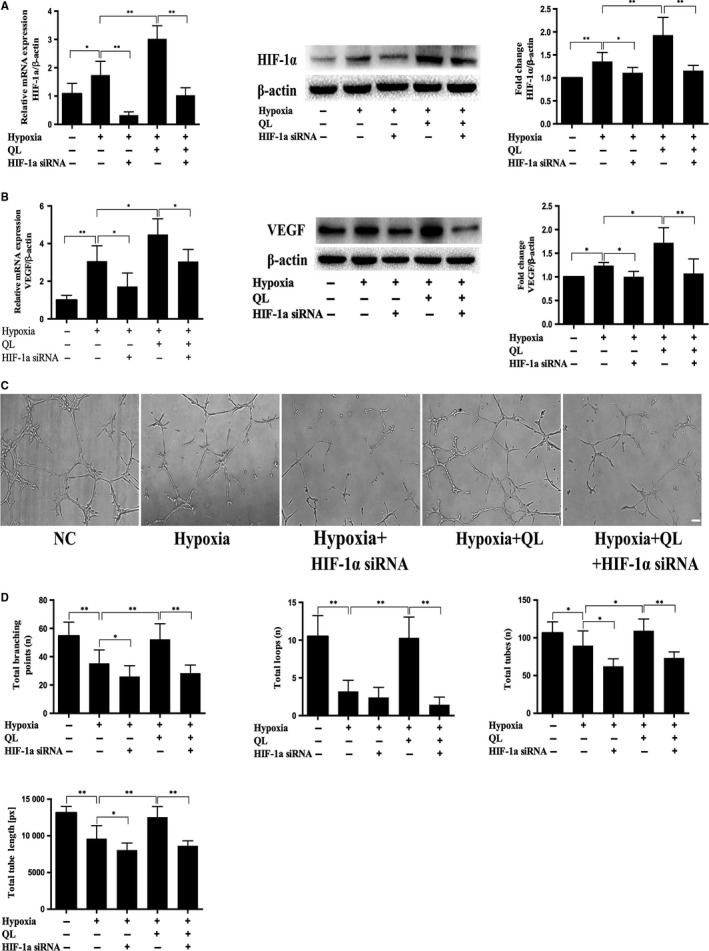
Effects of qiliqiangxin (QL) on cardiac microvascular endothelial cells (CMECs) angiogenesis under hypoxic condition. (A) QL increased the mRNA and protein level of HIF‐1α in hypoxic CMECs (n = 4‐6/group). (B) QL increased the mRNA and protein levels of VEGF in hypoxic CMECs (n = 4‐6/group). (C) QL treatment promoted tube formation of hypoxic CMECs (10×, Scale bar 100 μm) (n = 4/group). (D) Bar graphs of total branching points, loops, tubes and tube length (n = 4/group). Data were expressed as means ± SD. *P < .05; **P < .01

HIF‐1α has been reported to trigger angiogenesis, and VEGF, the most potent angiogenesis‐stimulating factor, is one of the major downstream effector of HIF‐1α. Firstly, our results showed that hypoxia exposure of CMECs increased mRNA and protein expressions of HIF‐1α and VEGF, which could be further up‐regulated by QL treatment, while HIF‐1α siRNA attenuated the beneficial effects of QL (Figure 3A,B). The level of VEGF secreted into the medium was also significantly increased after QL treatment (Figure S1D). We then used tube formation test to detect CMECs angiogenesis in vitro, by measuring the total loops, tubes, nets, branching points and tube length. The results showed that hypoxia exposure of CMECs impaired tube formation, as demonstrated by decreased number of total loops, tubes, branching points and tube length. Compared with hypoxia group, pre‐treatment with QL significantly promoted CMECs tube formation, which, however, were partly abolished by HIF‐1α siRNA (Figure 3C,D). These results indicated that QL could promote angiogenesis under hypoxic condition via HIF‐1α/VEGF pathway.

### 3.4 QL increased CMECs ATP production and improved CMECs glucose metabolism under hypoxic condition through glycolytic pathway

**Figure 4:**
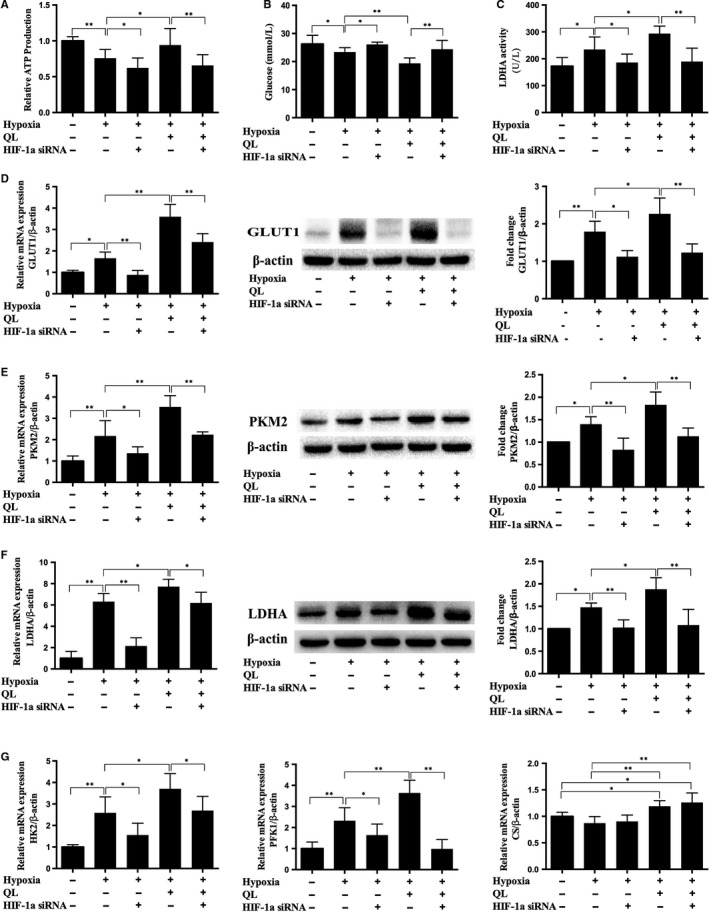
Effects of qiliqiangxin (QL) on glucose metabolism and ATP production. (A), (B) and (C). QL increased ATP production, decreased glucose level and promoted LDHA activity in hypoxic cardiac microvascular endothelial cells (CMECs) (n = 8‐11/group). (D), (E), (F) and (G). The mRNA and/or protein levels of GLUT1, HK2, PKM2, LDHA, PFK1 and CS(n = 4‐6/group). Data were expressed as means ± SD. *P < .05; **P < .01

ATPlite assay kit was used to detect ATP production, and the results showed that QL increased ATP production in hypoxic CMECs, while HIF‐1α siRNA could suppress this effect (Figure 4A), suggesting that QL could increase ATP production in hypoxic CMECs in a HIF‐1α‐dependent manner.

To examine the effect of QL on glucose metabolism of hypoxic CMECs, we collected cell culture supernatants after hypoxia exposure and detected glucose level and LDHA activity of various groups. The results showed that QL could decrease glucose level and increase LDHA activity through up‐regulating HIF‐1α activity (Figure 4B,C). We further examined the effects of QL on the key glycolysis‐related enzymes. As a result, hypoxia exposure could significantly up‐regulate the mRNA and/or protein expressions of glucose transporter 1 (GLUT1), hexokinase 2 (HK2), phosphofructokinase 1 (PFK1), pyruvate kinase M2 (PKM2) and lactic dehydrogenase (LDHA); pre‐treatment with QL could further up‐regulate their expressions, while was partly attenuated by HIF‐1α siRNA (Figure 4D‐G). These results indicated that QL could promote glycolysis and increase glucose uptake and ATP generation in hypoxic CMECs through up‐regulation of HIF‐1α and its downstream targets, especially the glycolysis‐related key enzymes.

We then examined glucose aerobic oxidation in CMECs, which is the dominant way of ATP generation in most cells. However, it is reported that only 1% glucose is metabolized in the Krebs cycle, and 99% goes to the glycolytic pathway in ischaemic endothelial cells.^17^ In this study, we found that, under hypoxic condition, the mRNA level of citrate synthase (CS), a key enzyme initiating Krebs cycle, did not significantly change. QL significantly up‐regulated its expression, while HIF‐1α siRNA had no effect, indicating that QL could enhance glucose aerobic glucose metabolism in endothelial cells exposed to hypoxia in a HIF‐1α‐independent pathway (Figure 4G).

### 3.5 AMPK/mTOR/HIF‐1α signalling pathway was involved in QL‐mediated induction of HIF‐1α and glycolysis in hypoxic CMECs

**Figure 5:**
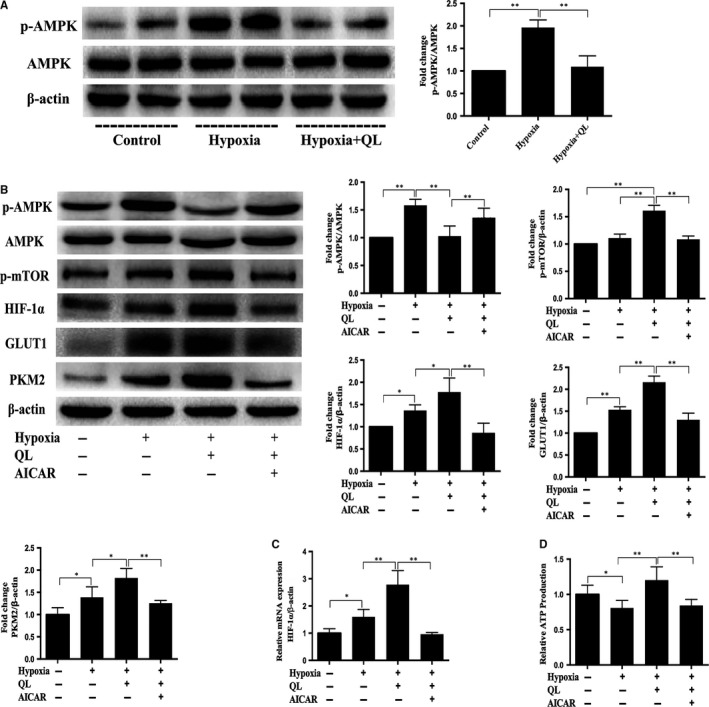
AMPK/mTOR/HIF‐1α pathway is involved in qiliqiangxin (QL)‐mediated HIF‐1α‐dependent glycolysis. (A) Cardiac microvascular endothelial cells (CMECs) were incubated with or without QL under hypoxia for 12 h, and QL decreased AMPK phosphorylation (n = 4/group). CMECs were incubated with or without QL and AICAR (1 mmol/L, AMPK activator) under hypoxia. (B) Protein expression of glycolysis key enzymes GLUT1 and PKM2 were determined by Western blot (n = 4/group). (C) HIF‐1α mRNA expression was measured by qRT‐PCR (n = 4/group). (D) After 12 h of hypoxia, ATP production was measured (n = 4/group). Data were expressed as means ± SD.*P < .05; **P < .01

It has been demonstrated that HIF‐1α could be regulated at translational level via AMPK/mTOR pathway under hypoxia. We thus evaluated the role of AMPK/mTOR in QL‐induced HIF‐1α protein accumulation in CMECs. Our data showed that QL treatment significantly decreased AMPK phosphorylation and increased ATP production, mTOR phosphorylation, GLUT1 and PKM2 expressions in CMECs. Moreover, activating AMPK with AICAR (1 mmol/L, AMPK activator) prior to QL treatment markedly reversed these effects (Figure 5A‐D). These data indicated that AMPK could affect the HIF‐1α activity and HIF‐1α‐associated anaerobic glycolysis.

### 3.6 QL inhibited the PHD3‐mediated HIF‐1α post‐translational hydroxylation in hypoxic CMECs

**Figure 6:**
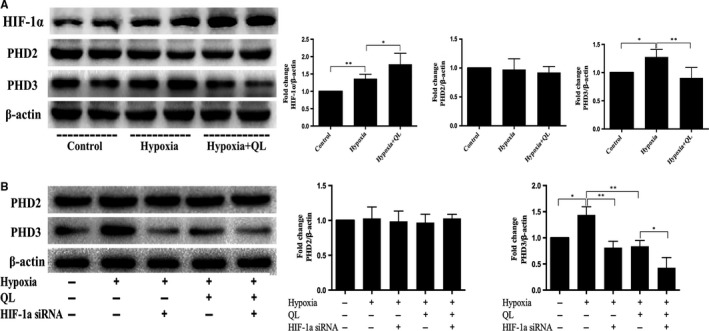
Effects of qiliqiangxin (QL) on HIF‐1α post‐translational hydroxylation. (A) QL increased HIF‐1α and decreased PHD3 expression, but have no effect on PHD2 expression in hypoxic cardiac microvascular endothelial cells (CMECs) (n = 4/group). (B) HIF‐1α enhanced PHD3 but not PHD2 expression of CMECs under hypoxia condition (n = 4/group). Data were expressed as means ± SD. *P < .05; **P < .01

To investigate the effect of QL on HIF‐1α post‐translational hydroxylation, we examined prolyl hydroxylase domain‐containing proteins (PHDs) expression, including PHD2 and PHD3. The increased HIF‐1α expression and decreased PHD3 expressions were observed in the Hypoxia+QL group, so we speculated that QL could improve HIF‐1α stabilization by inhibiting PHD3 instead of PHD2 (Figure 6A). Consistent with previous studies, we found that PHD3 expression was up‐regulated by HIF‐1α in hypoxia condition. In Hypoxia+QL+ HIF‐1α siRNA group, however, the inhibition of QL on PHD3 expression was more significant than the promotion of HIF‐1α (Figure 6B).

## 4 DISCUSSION

In the present study, we explore the protective effects and related mechanisms of QL on hypoxic CMECs in the absence or presence of HIF‐1α siRNA transfection. The results show that QL promotes endothelial cell proliferation and angiogenesis, inhibits apoptosis and ROS generation, restores polarized mitochondrial membrane potential and most importantly improves glucose metabolism in hypoxic CMECs via up‐regulation of HIF‐1α.

Normal cell metabolism depends on the regulatory delivery of fuel substrates and oxygen from vasculature to organs and tissues. CMECs, a special cell type derived from coronary microvessels, play a major role in promoting angiogenesis and delivering oxygen to myocardium. The fact that HIF‐1α is elevated under hypoxic conditions has been confirmed in most types of endothelial cells, for example, human/rat cardiac microvascular endothelial cells and human umbilical vein endothelial cells (HUVECs).^28^, ^29^, ^30^ As is known, activated HIF‐1α and its downstream effector VEGF are key regulators which can protect endothelial cells from hypoxia‐induced impairment and promote angiogenesis and cell migration.^7^ In the present study, however, with long‐time hypoxia, the protective mechanism initiated by adaptive HIF‐1α elevation seems decompensated and not sufficient. Our results showed that extreme hypoxia aggravated cell apoptosis by increasing ROS production and release, which caused hyperpermeability of the inner mitochondrial membrane and led to impaired proliferation and capillary‐like tube formation of CMECs. Previous studies also found that, with hypoxia time gradually increased, endothelial cells oxidative stress, apoptosis and autophagy also increased.^31^, ^32^ In CMECs hypoxia‐reoxygenation model, decreased cell viability, migration and angiogenesis were also observed, while HIF‐1a and VEGF expressions were elevated.^33^ Fortunately, in the present study, we found that QL remedy up‐regulated HIF‐1α and VEGF expressions, which directly facilitated adaptation and survival of endothelial cells exposed to hypoxia. Taken together, it can be concluded that QL promotes proliferation and angiogenesis of hypoxic CMECs through up‐regulating HIF‐1α/VEGF signalling.

Endothelium, located between blood and tissue, is an essential determinant element of glucose metabolism in various whole organs including brain and heart. The oxygen‐sensing transcription factor HIF‐1α is a key regulator of endothelial metabolism.^10^ GLUT1, one of the HIF‐1α downstream genes, is predominantly localized on plasma membrane and accounts for basal glucose uptake. GLUT1 is widely expressed in endothelial cells and is vital to enrichment and effective utilization of glucose in endothelial cells.^34^, ^35^ In the course of glycolysis, glucose molecules are taken up by GLUT1 and then phosphorylated to glucose‐6‐phosphate. PFK1 and PKM2 are the major rate‐limiting enzymes in glycolysis to produce pyruvate. LDHA catalyses the interconversion of pyruvate to lactate, which is the last step of glycolysis. To explore whether QL could improve glucose metabolism in hypoxic CMECs through regulating HIF‐1α and its downstream genes, we used siRNA to suppress HIF‐1α gene expression in QL‐treated hypoxic CMECs and investigated whether HIF‐1α knockdown could affect the expression levels of enzymes involved in glucose metabolism (GLUT1, HK2, PFK1, PKM2 and LDHA relating to the glycolysis; CS relating to Krebs cycle). The integrated analysis of RT‐PCR and Western blot demonstrated that the hypoxia‐induced expressions of glycolytic enzymes were further up‐regulated by QL in hypoxic CMECs, while this effect could be attenuated by HIF‐1α siRNA. Also, QL could increase LDHA activity and ATP production in hypoxic CMECs in a HIF‐1α‐dependent manner. Moreover, QL up‐regulated the Krebs cycle key enzyme (CS) expression under hypoxic condition in a HIF‐1α activity‐independent manner. Collectively, our results show that hypoxic CMECs mainly rely on glycolysis for ATP production, which can be enhanced by QL through up‐regulating HIF‐1α and its downstream genes.

The dependence of CMECs on glycolysis has multiple advantages. Firstly, most glycolytic key enzymes are located in the cytosol, which favours immediate ATP supplies for actin rearrangement to facilitate angiogenesis and vesicular secretion and generates required intermediates necessary for cell growth and migration.^36^, ^37^ In the present study, we confirmed that down‐regulated HIF‐1α and glycolytic key enzymes expressions in the Hypoxia+HIF‐1α siRNA group could further reduce proliferation and tube formation of hypoxic CMECs compared with Hypoxia group, while QL treatment up‐regulated HIF‐1α and glycolytic key enzymes expressions and promoted proliferation and angiogenesis of hypoxic CMECs. Secondly, despite being in direct contact with oxygen, CMECs still rely on glycolysis to generate energy, which can spare more oxygen and fatty acid to fuel cardiomyocytes, cardiac fibroblasts and other cardiac cell types. Thirdly, glycolysis can produce ATP faster and protect CMECs from the damage caused by mitochondrial electron leakage and ROS.^38^, ^39^ ΔΨm is by far the most accurate indicator of mitochondrial physiological function. Our data demonstrated a significant collapse of ΔΨm under hypoxic condition, as demonstrated by decreased ratio of aggregates/monomers corresponding to polarized and depolarized mitochondria, respectively. While QL intervention significantly elevated the ratio of aggregates/monomers compared with hypoxia group, such elevation was suppressed by HIF‐1α siRNA. Our results are in line with previous findings on the relationship between HIF‐1α and mitochondrial dysfunction.^40^, ^41^ It is known that ROS are mainly generated from complex III of the electron transport chain in the mitochondria, which are increased under hypoxic conditions.^42^ Activated HIF‐1α expression could prevent cancer cells from ROS‐induced apoptosis,^43^ while knockdown of HIF‐1α could induce cell apoptosis.^17^ In our study, we found that QL could decrease ROS production and caspase‐3 activity, increase the Bcl‐2/Bax ratio and inhibit cell apoptosis in a HIF‐1α‐dependent manner.

AMP‐activated protein kinase (AMPK) is a key sensor of AMP/ATP or ADP/ATP ratio and thus the cell energy level. It is activated under hypoxic condition to promote ATP consumption and/or inhibit ATP production.^44^ Previous researches found that AMPK could directly or indirectly suppress mTOR activity, thus limiting HIF‐1α protein synthesis.^45^, ^46^ In the present study, we found that QL significantly inhibited AMPK phosphorylation. To investigate whether this inactivation of AMPK contributes to the up‐regulation of mTOR activity, which subsequently increases HIF‐1α mRNA translation, we treated CMECs with AICAR (AMPK activator). The results showed that AICAR effectively inhibited mTOR activity and reduced HIF‐1α activity, indicating that increased HIF‐1α activity in hypoxic CMECs may be regulated by the AMPK/mTOR pathway. To investigate whether AMPK/mTOR/HIF‐1α axis is involved in elevated anaerobic glycolysis in hypoxic CMECs, the ATP production and glycolytic key enzymes (GLUT1 and PKM2) protein expressions were detected by Western blot analysis after treatment with or without QL and AICAR. It is notable that AICAR activated AMPK/mTOR/HIF‐1α axis, while led to down‐regulation of ATP production, GLUT1 and PKM2 protein level. Therefore, these data suggest that QL can promote glycolysis of hypoxic CMECs via regulating AMPK/mTOR/HIF‐1α pathway.

Then, we further observed the effect of QL on HIF‐1α stabilization. Under aerobic conditions, HIF‐1α is post‐translational hydroxylated within the central oxygen‐dependent degradation domain at 1 or 2 prolyl residues. Prolyl hydroxylase domain‐containing proteins (PHDs) are negative regulators of the hypoxia‐inducible factors (HIFs). Three human HIF‐1α prolyl hydroxylases (PHDs) that can hydroxylate the prolyl residues of HIF‐1α have been identified. PHD2 and PHD3 have been proved to be regulators of HIF‐1α, which act as HIF‐1α repressors in hypoxia and avoid excessive HIF‐1α gene expressions.^47^ PHD2 is the main regulator of HIF‐1α under normoxia and hypoxia, while PHD3 might regulate HIF‐1α in more severe and prolonged hypoxia, PHD2 and PHD3 expressions have been shown to be up‐regulated by HIF‐1α and other protein interactions in hypoxia condition.^48^ In our study, we found that PHD3 protein expression was increased in Hypoxia group, and QL treatment significantly decreased its expression and increased HIF‐1α expression, while PHD2 expression showed no difference in 3 groups. These results indicated that QL promoted HIF‐1α accumulation by inhibiting PHD3 expression. We then detected the effect of HIF‐1α on PHDs expression in hypoxia CMECs. As a result, HIF‐1α did not impact on PHD2 expression. However, it significantly induced PHD3 expression under Hypoxia group, PHD3 expression was significantly attenuated in Hypoxia + HIF‐1α siRNA group, and the inhibitory effect of QL on PHD3 was more significant than HIF‐1α promotional effect in the Hypoxia + QL group.

In conclusion, our results indicate that QL can protect CMECs against hypoxic injury by promoting angiogenesis and proliferation, reducing ROS generation and apoptosis and increasing polarized mitochondrial membrane potential and ATP production. The beneficial effects of QL are mainly mediated by up‐regulating HIF‐1α activity and glycolysis. We also demonstrated that QL promotes the mRNA and protein expressions of HIF‐1α in hypoxic CMECs by regulating AMPK/mTOR/HIF‐1α pathway and deduced that QL maintains HIF‐1α stabilization by decreasing PHD3 expression.

## CONFLICT OF INTEREST

The authors confirm that there is no conflict of interests.
