# Supplementary figures and images for: Qiliqiangxin attenuates hypoxia‐induced injury in primary rat cardiac microvascular endothelial cells via promoting HIF‐1α‐dependent glycolysis
Source: J Cell Mol Med. 2018 Mar 4;22(5):2791–803. doi: 10.1111/jcmm.13572 (PMC5908112; doi:10.1111/jcmm.13572)

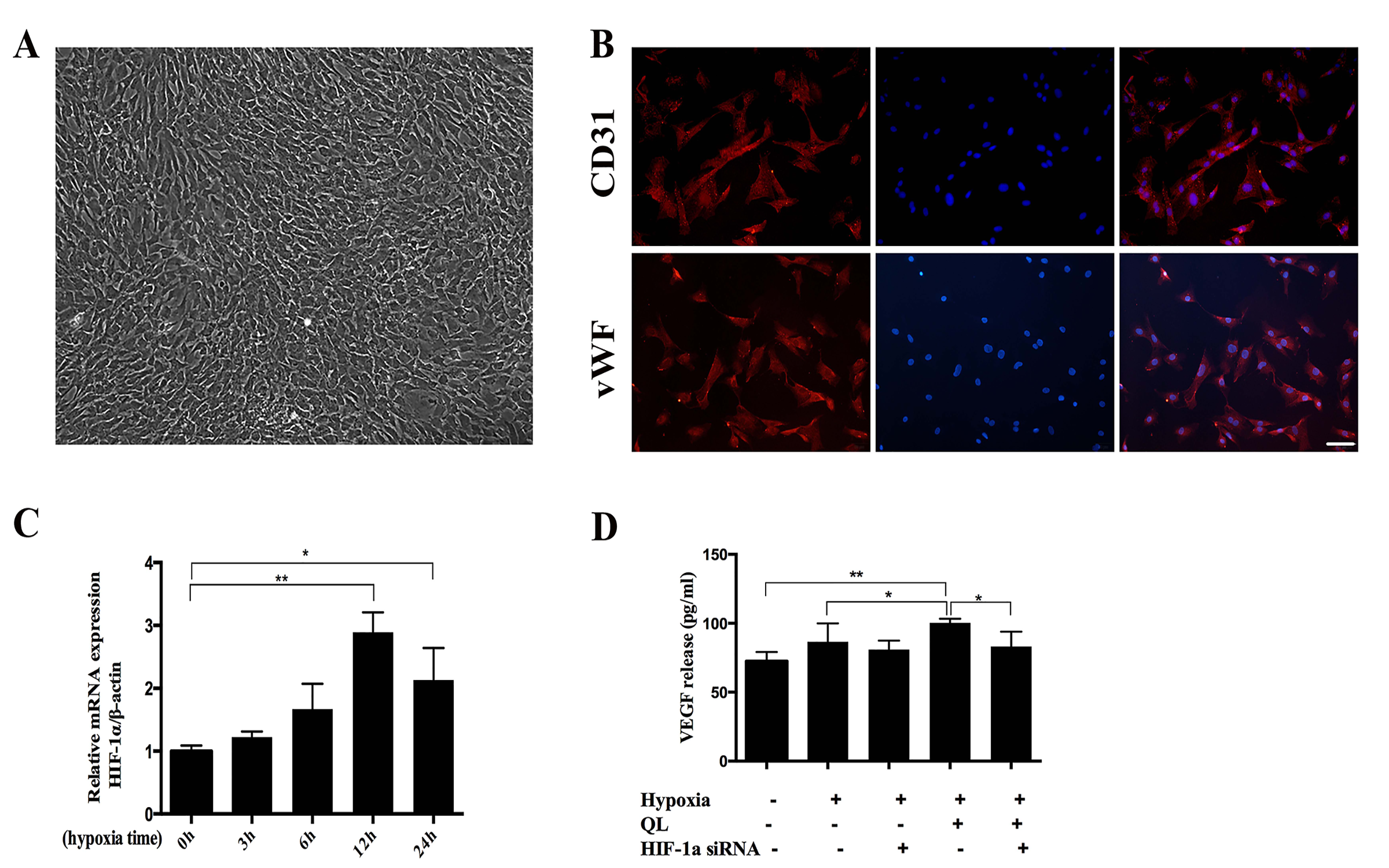

Supplement: Supplementary file 1 [file JCMM-22-2791-s001.tif]
